# High-Density Mineralized Protrusions and Central Osteophytes: Associated Osteochondral Junction Abnormalities in Osteoarthritis

**DOI:** 10.3390/diagnostics10121051

**Published:** 2020-12-05

**Authors:** Alecio F. Lombardi, Qingbo Tang, Jonathan H. Wong, Judith L. Williams, Saeed Jerban, Yajun Ma, Hyungseok Jang, Jiang Du, Eric Y. Chang

**Affiliations:** 1 Research Service, VA San Diego Healthcare System, San Diego, CA 92161, USA; q1tang@health.ucsd.edu (Q.T.); jhw033@health.ucsd.edu (J.H.W.); judydrj.jw@gmail.com (J.L.W.); sjerban@health.ucsd.edu (S.J.); yam013@health.ucsd.edu (Y.M.); h4jang@health.ucsd.edu (H.J.); jiangdu@health.ucsd.edu (J.D.); ericchangmd@gmail.com (E.Y.C.); 2 Department of Radiology, University of California, San Diego, CA 92122, USA

**Keywords:** HDMP, central osteophytes, osteochondral junction, osteoarthritis

## Abstract

The aim of this study was to determine the association between high-density mineralized protrusions (HDMPs) and central osteophytes (COs), and describe the varying appearance of these lesions using advanced clinical imaging and a novel histological protocol. Seventeen consecutive patients with clinically advanced knee osteoarthritis undergoing knee arthroplasty were included. Surgical tissues containing the osteochondral region were investigated using computed tomography (CT); a subset was evaluated using confocal microscopy with fluorescence. Tissues from seven subjects (41.2%) contained HDMPs, and tissues from seven subjects (41.2%) contained COs. A significant association between HDMPs and COs was present (*p* = 0.003), with 6 subjects (35.2%) demonstrating both lesions. In total, 30 HDMPs were found, most commonly at the posterior medial femoral condyle (13/30, 43%), and 19 COs were found, most commonly at the trochlea (5/19, 26.3%). The HDMPs had high vascularity at their bases in cartilaginous areas (14/20, 70%), while the surrounding areas had elevated levels of long vascular channels penetrating beyond the zone of calcified cartilage (*p* = 0.012) compared to HDMP-free areas. Both COs and HDMPs had noticeable bone-resorbing osteoclasts amassing at the osteochondral junction and in vascular channels entering cartilage. In conclusion, HDMPs and COs are associated lesions in patients with advanced knee osteoarthritis, sharing similar histologic features, including increased vascularization and metabolic bone activity at the osteochondral junction. Future studies are needed to determine the relationship of these lesions with osteoarthritis progression and symptomatology.

## 1. Introduction

Osteoarthritis (OA) is the most common form of arthritis and is a leading cause of disability worldwide [1]. Classically, OA has been considered a primary disorder of articular cartilage, but it is now well-known that OA is, in fact, a multi-faceted disease with various etiologies. Abnormalities or lesions of any of the of joint components like bone [2], meniscus in the knee [3], ligaments [4], fat pads [5], and synovium tissue [6,7] may contribute both to the pathogenesis and symptomatology of OA.

Recently, there has been increasing interest and awareness of the importance of the osteochondral junction (OCJ), particularly in early OA [8]. Abnormalities at the OCJ include loss of integrity and increasing degrees of plasticity [9]. Integrity loss may result from a mechanical fracture of the subchondral bone and calcified cartilage, either accompanied by a fracture of the overlying non-calcified cartilage, or isolated with a preserved articular surface [10]. Plasticity is a more gradual, cellular process where upregulation of growth factors results in channels formed by osteoclasts and chondroclasts leading to potential neurovascular invasion [11]. These abnormalities may not be mutually exclusive processes since micro-cracks associated with integrity loss may trigger cellular processes [12], and plasticity in the OCJ may be associated with focal cartilage degeneration [13,14,15]. 

Central osteophytes (COs) are commonly found in osteoarthritic knees as flat, column-, or button-shaped bone exostoses in areas with relatively preserved cartilage, and are believed to be caused by endochondral ossification at the OCJ, but their pathogenesis is not completely understood [16,17,18].

In 2003, Ferguson et al. described an acellular hyper-dense dystrophic calcification occurring in the calcified layer of human articular cartilage [19]. These were later termed “high-density mineralized protrusions” (HDMPs), which form because of micro-cracks in the subchondral bone plate and the calcified layer of cartilage [20]. HDMPs may damage and extend far into the non-calcified articular cartilage [21]. Of note, HDMPs are notoriously difficult to identify using conventional microscopic techniques, such as decalcification with paraffin embedding, and were initially discovered using backscattered scanning electron microscopy [19]. HDMPs have been shown to fragment and produce sharp-edged particles, at times extruding into the joint space [22]. Beyond this, however, the origin and fate of HDMPs are largely unknown. It has been speculated that HDMPs may be related to COs [20,21,23], but to date, this has not been formally investigated. Recent evidence corroborated central osteophytes are highly prevalent in osteoarthritic patients [24]. Thus, the purpose of our study was to determine the association between HDMPs and COs, and describe the various appearances of these lesions using advanced clinical imaging and a novel histological protocol. 

## 2. Materials and Methods

### 2.1. Study Population and Surgical Tissues

This study was approved by our Institutional Review Board and all subjects provided signed informed consent. All the procedures were carried out following the rules of the Declaration of Helsinki of 1975, revised in 2013. Consecutive participants with clinically advanced knee osteoarthritis undergoing knee arthroplasty were enrolled. Excised osteochondral tissues from the surgery were collected and divided into eight anatomic regions, including: patella, trochlea, medial tibial plateau, lateral tibial plateau, weight-bearing medial femoral condyle, posterior medial femoral condyle, weight-bearing lateral femoral condyle, and posterior lateral femoral condyle. Immediately after surgery, tissues were fixed with 2% paraformaldehyde/12.5% of saturated picric acid solution in 0.1 M phosphate buffer (pH 7.0) at 4 °C for 24 h, then rinsed with saline and stored at 4 °C until imaging. 

### 2.2. Computed Tomography (CT) Imaging and Analysis

All tissues were scanned on a third-generation, dual-source, multidetector CT scanner (Siemens SOMATOM Force; Siemens Healthcare AG, Erlangen, Germany) at isocenter using the ultra-high-resolution (UHR) mode, 120 kVp, 90 mA, and 0.3 pitch. Images were reconstructed using the Ur77u kernel with Edge Technology at 0.1 mm isotropic resolution, but true achievable resolution is 0.2 mm (25 Lp/cm at 10% modulation transfer function, MTF) in the x-y plane and 0.24 mm (21 Lp/cm at 10% MTF) in the z-plane [25]. For tissues obtained from a single patient, imaging times were typically less than 30 s. 

Using a DICOM viewer (RadiAnt 2020.1; Medixant, Poznan, Poland), multiplanar images were reconstructed and independently evaluated by two fellowship-trained musculoskeletal radiologists (A.F.L. and E.Y.C., with 7 and 9 years of experience, respectively) for the presence or absence of HDMPs or COs. HDMPs were defined as high-density well-defined calcifications emerging from the OCJ towards the articular surface (11). HDMPs have a distinct clinical imaging appearance compared with calcium pyrophosphate dihydrate crystal deposition, which is most often located in the intermediate layer of cartilage [26]. COs were defined as extrusions of subchondral bone beyond the osteochondral junction in a more central location than the peripheral margins of the articular cartilage. COs have a characteristic appearance with a flat surface, sharply demarcated step-off at their margins, and are often button-shaped [27]. 

After identification of each HDMP, the following criteria were determined: (1) morphology, including dimensions (height × width × length in mm), (2) attenuation (in Hounsfield units, HU), (3) presence of COs in the same anatomic region (yes or no), (4) presence of COs in a different anatomic region (yes or no), and (5) distance from the HDMP to the CO (in mm), if in the same region. For each identified HDMP and CO, subchondral bone plate attenuation (HU) was also measured at the lesion and at an adjacent normal-appearing region without discernable abnormality.

### 2.3. Fluorescent Labelling

After CT imaging, tissues were rinsed with saline and stored in an antifreeze solution containing 30% ethylene glycol, 30% sucrose, and 1% polyvinylpyrrolidone at −20 °C until further processing. Bone sections of 1–2 mm thickness were cut with a precision sectioning saw (IsoMet 1000; Buehler, IL, USA). Sections were decalcified with 10% ethylenediaminetetraacetic acid (pH 7) at 4 °C for up to 10 days. Radiography (Trident Specimen Radiography System; Hologic, Marlborough, MA, USA) of the sections was used as quality control to confirm the localization of HDMPs and COs.

Fluorescence-based staining was employed on a subset of tissues to detect osteoclasts [28] and vascular cells. Specifically, in order to detect tartrate-resistant alkaline phosphatase (TRAP) activity, sections were washed with saline, then incubated overnight with 50 mM CaCl_2_, MgCl_2_, and MnCl_2_ in Tris-buffered saline (pH 7.4) at 4 °C. Next, sections were rinsed with saline and incubated in a solution containing 200 mM acetate, 50 mM tartrate (pH 5.0), and 5 µM ELF-97 (ThermoFisher Scientific, Waltham, MA, USA) for 30 min at 4 °C, then an additional 60 min at room temperature. The enzymatic reaction was stopped by washing with phosphate-buffered saline (PBS). To detect vascular cells, sections were washed with tris(hydroxymethyl)aminomethane-buffered saline (TBS) and incubated overnight at 4 °C with 4 µg/mL Dylight 649 conjugated *Ulex europaeus* Agglutinin I (UEA-I 649) (Vector Laboratories, Burlingame, CA, USA) and 0.2 µg/mL 4′,6-diamidino-2-phenylindole (DAPI) in TBS supplemented with 2 mM CaCl_2_ and MgCl_2_. Sections were washed with saline and stored in antifreeze at −20 °C until imaging. 

### 2.4. Confocal Imaging and Histologic Analysis 

Fluorescence was imaged with an inverted confocal laser scanning microscope (LSM880; Zeiss, Germany) using a 10× objective. Stained sections were placed in antifreeze on a #1 coverslip glued to the bottom of a cut plastic dish. The following excitation lasers were used: DAPI, 405 nm; ELF-97, 405 nm; UEA-I 649, 633 nm. Epifluorescence was collected based on spectra of the three fluorophores: DAPI, 415–460 nm; ELF-97, 541–552 nm; UEA-I 649, >645 nm. Multiple tile scan images along the z-planes were generated to cover the whole sections with area up to 5 cm^2^, depth up to 250 µm, and resolution of 2.77 × 2.77 µm/pixel. Imaging times per section were typically less than 60 min. Images were processed, and stacks of images were projected using imaging software (ZEN lite; Zeiss, Oberkochen, Germany and ImageJ; NIH, Bethesda, ML, USA). 

A subset of tissues was selected for histological analysis based on the CT morphology of HDMPs and COs, to include all the different aspects within the spectrum of presentations. HDMP and CO regions were assessed for the presence and extent of vascular invasion and osteoclast activity. First, each HDMP was surveyed across the z-stack and scored for the presence of UEA-stained blood vessels contacting the protrusion area. Second, within a 2-mm region centered on the HDMP, tidemarks were observed and counted, and vascular channels emanating from the subchondral marrow and reaching the OCJ were counted (channel density, expressed as channels per mm). Each channel’s maximum extent was determined to be in the subchondral bone at the OCJ, in the zone of calcified cartilage (cartilage below tidemark), or in the uncalcified cartilage above the most superficial tidemark observed.

For each area containing HDMPs, one to two HDMP-free areas of 2 mm width, located >2 mm from any HDMPs but within the same section if possible, were chosen as control areas, and channels were counted in the same way as the HDMP-containing regions. All histologic analyses were performed by a histotechnician (J.H.W., 18 years of experience in histopathology), who was blinded to the radiologic results. 

### 2.5. Statistical Analysis

All statistical analyses were performed using R (version 4.0.2; R Development Core Team, 2020, Vienna, Austria) and RStudio (version 1.3.959). The Shapiro–Wilk test was used to assess normality. Descriptive statistics were performed. Fisher’s exact test was used to determine the association between the presence of HDMPs and COs among subjects. Means were compared using one-way ANOVA or Student’s *t*-tests as appropriate. Tukey’s post hoc analysis was performed to adjust the *p*-value of each pair-wise comparison. *p* < 0.05 was considered to represent significant findings.

## 3. Results

Seventeen male subjects were included in this study (67.9 ± 7.4 years-old; mean ± standard deviation) with 16 total knee arthroplasties and one unicompartmental knee arthroplasty. A brief description of the patient demographics and clinical data is provided in Table 1.

### 3.1. Radiologic Analysis

Both readers were fully concordant in the identification of HDMPs and COs on the CT images. In total, tissues from seven subjects (41.2%) contained HDMPs and tissues from seven subjects (41.2%) contained COs. A statistically significant association between HDMPs and COs was present (*p* = 0.003), with six subjects (35.2%) demonstrating both HDMPs and COs. Tissues from nine patients demonstrated neither HDMPs nor COs.

Considering all available tissues, we found 30 HDMPs, with anywhere between one and 16 appearing in a single joint. HDMPs were present in all anatomic regions but were most common at the posterior medial femoral condyle (13/30, 43%), followed by the posterior lateral femoral condyle (6/30, 20%). 

Again, considering all available tissues, we found 19 COs, with anywhere between one and six appearing in a single joint. As was the case with HDMPS, COs were present in all anatomic regions but were most common at the trochlea (5/19, 26.3%), followed by the posterior lateral femoral condyle (5/19, 26.3%). A description of the distribution of HDMPs and COs among patients and in which patients both lesions were present can be seen in Table 2.

**Figure 1:**
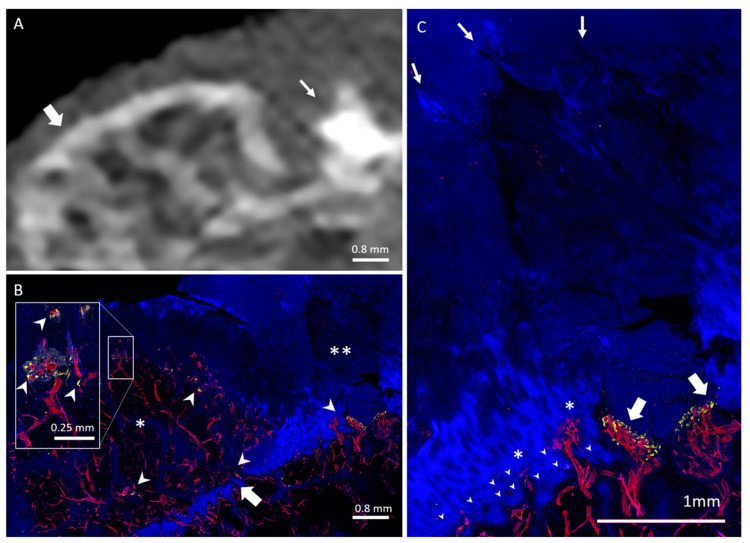
Weight-bearing lateral femoral condyle containing a CO and an HDMP. (**A**) CT image shows a column shaped HDMP (thin arrow) with sharp superficial margins, next to a CO (thick arrow) which presents with the original subchondral bone plate at its base. Thickening and irregularity of the subchondral bone plate under the HDMP is observed. (**B**) Micrograph shows the CO (asterisk) and HDMP (double asterisk), with osteoclasts (TRAP activity, yellow) in the marrow, OCJ, and vascular channels at both lesions (arrowheads). Vessels (UEA-I, red) fill the marrow and channels, and traverse the remaining OCJ on the deep surface of the CO (thick arrow). (**C**) Enlarged view of the HDMP, with needle-puncture-like extensions of the upper margin (small arrows), and strong TRAP activity (yellow) at the deep margin (thick arrows). Both reduplicated tidemarks (arrowheads) are breached by vascular channels (asterisks).

**Figure 2:**
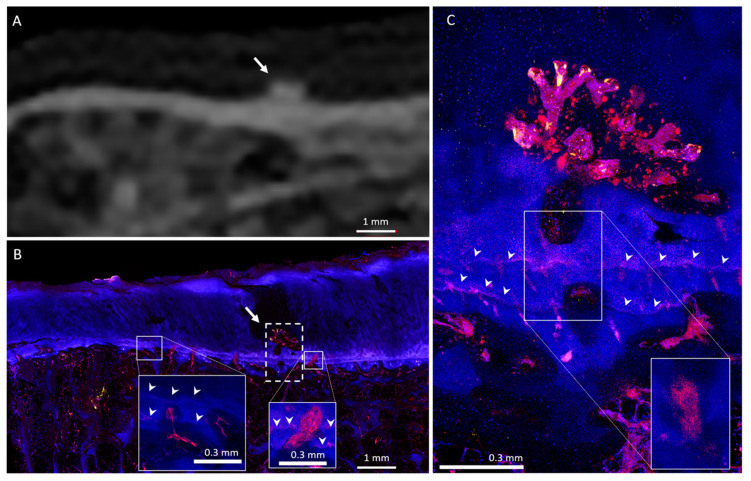
Medial tibial plateau containing a small HDMP. (**A**) CT image shows mesa-like HDMP (arrow) with associated subchondral bone plate thickening. (**B**) Micrograph in the same location as Figure 2A shows HDMP associated with the calcified cartilage (arrow), exhibiting superficial vascular invasion and cartilage degeneration in the dotted-boxed area. Vascular channels are present at the OCJ throughout the tissue (e.g., solid boxed and inset). Distant from the HDMP, channels tend to terminate within the calcified cartilage, but near the HDMP, they more frequently cross all tidemarks (arrowheads). Insets are subsets of the full z-stack of confocal optical planes, which allows identification of some channels not otherwise apparent in a full-stack maximum-intensity projection (large image). (**C**) Enlarged image of the boxed area in Figure 2B. Fluorescent protrusion extends above both tidemarks (arrowheads) and is highly vascularized at its superficial margin, where endothelial (UEA-I, red), TRAP-positive (TRAP activity, yellow), and other cells (DAPI-stained nuclei, blue) form organized structures. The boxed area shows the inset at the same scale, as observed within a subset of optical planes, revealing the uninterrupted tortuous path of the large vascular channel leading to the HDMP.

**Figure 3:**
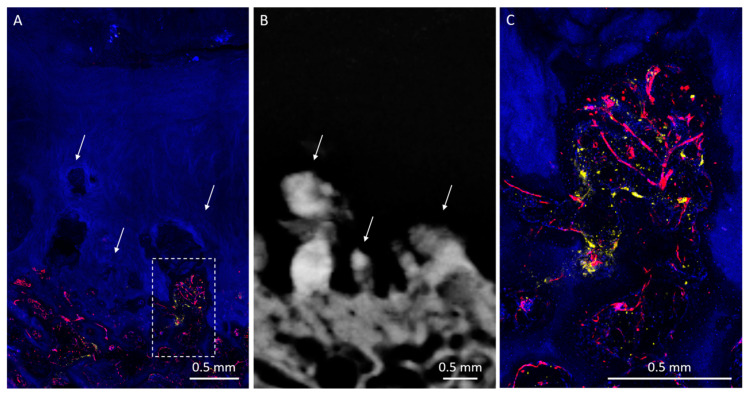
Posterior medial femoral condyle with amorphous HDMPs. (**A**) Micrograph shows richly vascularized OCJ with overlying hypointense regions within diseased cartilage (arrows). Enlargement of the boxed area shown in Figure 3C. (**B**) Radiograph shows irregularly shaped fragmenting HDMPs (arrows) associated with a thickened and irregular subchondral bone plate. Some HDMP fragments appear needle shaped. (**C**) Enlarged image of boxed area in Figure 3A. The base of the HDMP is highly vascularized (UEA-I, red) and demonstrates strong TRAP activity (yellow). Many cell nuclei (DAPI, blue) are present in this region, as are bone trabeculae.

**Figure 4:**
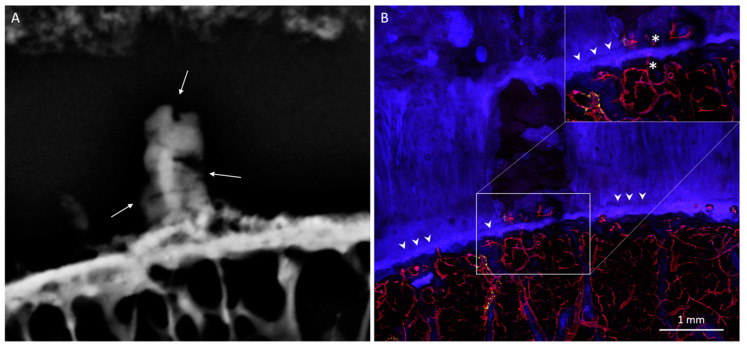
Posterior lateral femoral condyle with HDMP. (**A**) Radiograph shows column-shaped HDMP with a mesa-like base, presenting with several layers of mineralization and fragmenting areas (thin arrows). (**B**) Micrograph of the same anatomic region as Figure 4A. The base of the HDMP (enlarged, inset) is vascularized at locations that interrupt the tidemark (arrowheads). When surveying individual confocal optical planes within the z-stack (not shown), vessels (UEA-I, red) are seen to traverse the OCJ (at asterisks) from the marrow.

**Figure 5:**
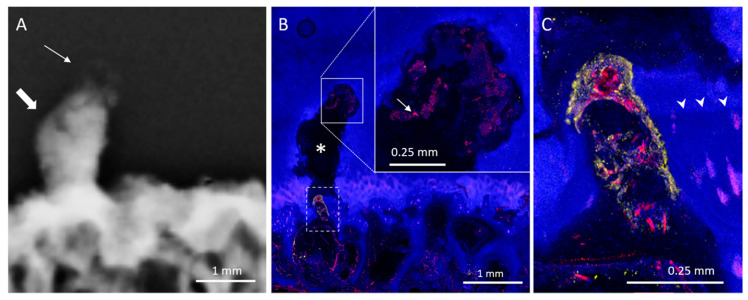
Posterior lateral femoral condyle containing a fragmented HDMP. (**A**) Radiograph shows irregularly shaped HDMP (thick arrow) with fragmented appearance and severely eroded superficial margin (thin arrow). (**B**) Micrograph shows that contents were not preserved in the core of the HDMP (asterisk), but of the remaining material, the detritus in the superficial tip (inset) was weakly positive for UEA-I. Some intense, non-diffuse, curvilinear staining was also present (arrow), characteristic of vessels. (**C**) Enlarged image of dotted-boxed area from Figure 5B shows many TRAP-expressing cells (yellow) lining a large vascular channel which extends through bone and tidemark (arrowheads) to reach the base of the HDMP.

HDMPs resembled a variety of shapes, such as a column (Figure 1), button (Figure 2), or egg, while others were more amorphous (Figure 3). Some HDMPs had several layers of mineralized material with different densities (Figure 4). Of the 30 HDMPs, 15 (50%) showed signs of fragmentation, either at the base or apex. The fragments had irregular margins (Figure 5) and, at times, were needle-shaped (Figure 1). Two HDMPs were in an oblique orientation (approximately 45°) relative to the articular surface.

Mean HDMP height (1.4 ± 0.7 mm) was greater than mean width (0.9 ± 0.3 mm) (*p* = 0.001) and mean length (0.8 ± 0.3 mm) (*p* < 0.001). Mean HDMP attenuation measured 2294 ± 428 HU. Mean maximum subchondral bone plate attenuation beneath the HDMPs (1839 ± 211 HU) and at COs (1717 ± 163 HU) was greater than that of adjacent normal-appearing regions (936 ± 116 HU) (*p* < 0.001). No significant difference between subchondral bone plate attenuation was observed beneath HDMPs and at COs (*p* = 0.11). Of the six subjects who presented with both HDMPs and COs, five demonstrated both types of lesions in the same anatomic knee region. The distance between lesions ranged from 0 (i.e., HDMP originated from the central osteophyte) to 36 mm. 

**Figure 6:**
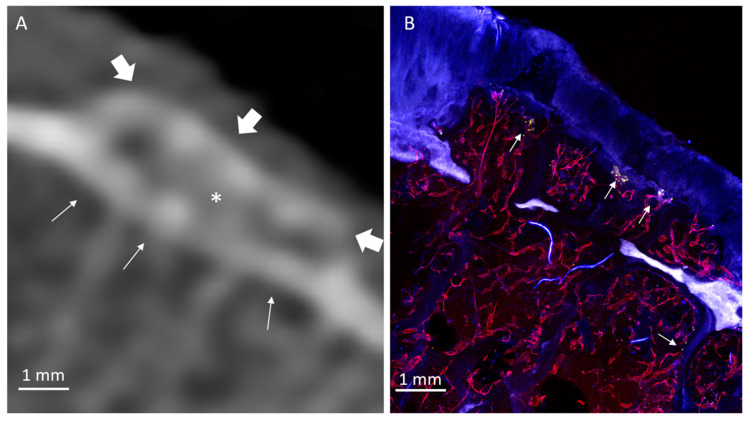
Posterior lateral femoral condyle with CO. (**A**) CT image shows a mesa-like CO (asterisk) with reduplicated subchondral bone plate at the superficial margin (thick arrows) and irregular remnant subchondral bone plate seen across the base (thin arrows). (**B**) Corresponding micrograph shows the CO overlying the discontinuous remnant of the previous subchondral bone plate. Within the CO is highly vascular (UEA-I, red) marrow, with bone resorption (TRAP activity, yellow) at the osseus margin.

**Figure 7:**
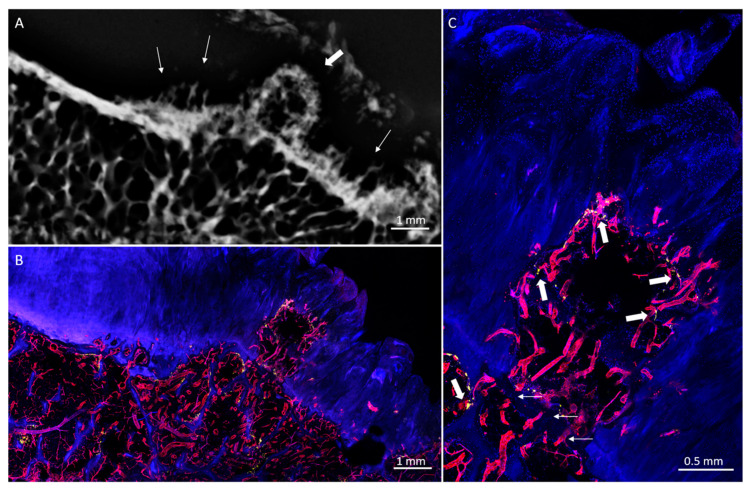
Posterior lateral femoral condyle with CO. (**A**) Radiograph shows a column-shaped CO arising from a marginal osteophyte (thick arrow). Flame-shaped cartilage calcifications are seen adjacent to the CO (thin arrows). (**B**) Corresponding micrograph shows fibrillated cartilage surrounding a highly vascularized (UEA-I, red) projection extending superficially from the marginal osteophyte. (**C**) Vessels traverse the OCJ at the base of the CO (thin arrows). TRAP activity (yellow, thick arrows) is present within the CO and in the marginal osteophyte below it.

COs demonstrated a characteristic mesa, or button-like, shape with a flat or irregular subchondral bone plate (Figure 1, Figure 6 and Figure 7). Of the 19 COs found, evidence of the original subchondral bone plate was present in 12 (63.1%). The remnant bone plate extending across the base of the CO could appear intact or discontinuous (Figure 6 and Figure 7). Mean CO length (6.8 ± 4.3 mm) and mean width (5 ± 2.2 mm) were greater than mean height (2.3 ± 0.9 mm) (*p* < 0.001). No significant difference was observed between mean CO length and width (*p* = 0.13).

### 3.2. Histological Analysis

**Figure 8:**
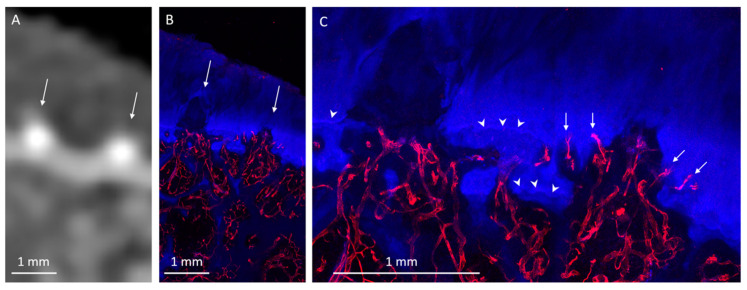
Posterior lateral femoral condyle with HDMPs. (**A**) CT image shows small egg-shaped HDMPs (thin arrows). (**B**) Corresponding microscopy, with many blood vessels (UEA-I, red) supplying the bases of the HDMPs. (**C**) Enlarged view shows the HDMPs extending from the OCJ superficially past the duplicated tidemark. Near the HDMPs, vascular channels (arrows) tend to breach the most superficial of the reduplicated tidemarks (arrowheads).

Twenty HDMPs covering the spectrum of presentations were selected for histological analysis. HDMPs had vascularization at the base in 70% (14/20) of cases. Vasculature was observed, through the thickness of the confocal z-stack, to travel from the marrow space through the OCJ to the base of the HDMPs. TRAP activity was common near the OCJ, including inside vascular channels near the HDMPs (Figure 1). Total density of vascular channels was lower in HDMP-containing areas compared with HDMP-free areas (means 2.5 and 3.3 per mm, *p* = 0.012). However, the density of channels that crossed the tidemark to reach the uncalcified cartilage in HDMP-containing areas was higher compared with HDMP-free areas (means 1.1 and 0.4 per mm, *p* = 0.021) (Figure 2 and Figure 8). Since the density of channels entering the cartilage to any depth was not different in the two types of areas (*p* = 0.634), the increase in long channels in the HDMP-containing areas was balanced by a concomitant decrease in short cartilage channels that did not cross the tidemark (means 0.7 and 1.4 per mm, *p* = 0.015).

COs were observed to be filled with highly vascularized marrow (Figure 1, Figure 6 and Figure 7). The remnant of the pre-existing bone plate was often visible in the trabecular structure of the deep aspect of the CO. CO marrow, and especially the OCJ at the superficial surface of the COs, were often strongly populated by large TRAP-positive osteoclasts (Figure 1, Figure 6 and Figure 7). These osteoclasts were often found in the cartilage inside vascular channels originating from the CO marrow space. 

Considering all types of areas and not separated according to lesion content, we observed vascular channels near the osteochondral junction with a mean spatial density of 2.8 ± 0.79 per mm. Channels that entered the cartilage, but were not observed to cross the tidemark, occurred at 0.98 ± 0.69 per mm, while those that crossed the tidemark to reach uncalcified cartilage were 0.85 ± 0.77 per mm. 

## 4. Discussion

In this study, we found that HDMPs and COs are associated lesions and concurrently present in 35% of subjects with advanced knee osteoarthritis. Overall, both lesions were common in our cohort, with 41% prevalence of HDMPs and 41% prevalence of COs. Our results are comparable to Thomas et al., who found an HDMP prevalence of 38% in 13 cadaveric knees when confirmed using CT imaging. Abrahim-Zadeh et al. also found a prevalence of 41% of knee COs in their study of 133 specimens [27]. Lower CO prevalence in the range of 14–18% has been shown with in vivo studies on younger patients [15,27,29]. In a cohort of 400 subjects with no, doubtful, or mild signs of radiographic OA from the Osteoarthritis Initiative, Kretzschmar et al. found a 7% incidence of COs over six years [14]. A more recent case-control study showed a higher prevalence and increased size of central osteophytes among OA patients but also more than the expected prevalence among controls, increasing the impression of temporality and a “dose–response” relationship between COs and OA [24]. Although these results demonstrate that COs develop over time with advancing age, it is interesting that they seldom, if ever, appear on denuded joint surfaces [16]. 

The microscopic appearances of these lesions have been studied before, particularly in COs. Half a century ago, Henry Jaffe described two histologic appearances of “flat exostoses” (now more commonly referred to as COs), including one where a single calcified cartilage zone appears “shifted” towards the articular surface, and a second where the calcified cartilage zone appears “reduplicated” (i.e., the original subchondral bone plate is visible across the base of the CO) [16]. In some “reduplicated” cases, the original subchondral bone plate eventually disappears, and the end result is the “shifted” CO appearance [16]. All three types of COs (shifted, reduplicated, and intermediate) were seen in our study. Pritzker et al. later described the process leading to COs, with an initial microfracture of the subchondral bone plate, followed by fibrocartilaginous proliferation, and subsequently osseous metaplasia to form an osteophyte [17]. 

HDMPs were first described in humans and characterized by Ferguson et al. [19] and Boyde et al. [20,21,22]. The inciting event is a microfracture of the osteochondral junction with matrix extrusion through the subchondral bone plate [30]. Mineralization follows, which is of such hardness that it is second only to dental enamel within the human body [20]. These hyperdense areas fragment and produce hard sharp-edged particles that destroy the surrounding cartilage [19]. To date, HDMPs have only been described as acellular hyperdense dystrophic calcifications. Our results now show that HDMPs demonstrate a high prevalence of vascular invasion, originating from subchondral bone and advancing through osteochondral tubular channels. Like previous studies, we note that HDMPs may fragment. Furthermore, we show that increased bone turnover or metabolic activity at the base of HDMPs accompany the advancing vascular front, similar in appearance to developed COs. 

It is worthwhile to note that HDMPs were an unknown entity until less than two decades ago. From the histological perspective, two reasons for their previous obscurity are: (1) loss of the highly mineralized matrix during decalcification and paraffin embedding, with subsequent misinterpretation of the split tissue as sectioning artifact, or (2) in cases where demineralization is incomplete, loss of the hyperdense focus during the sectioning process [21]. From the clinical imaging perspective, it is likely they were “hiding in plain sight.” Their presence may also be suggested using high-resolution MRI at 0.20–0.23 mm isotropic resolution [20,31] and we now show that they may be more readily seen and identified using a recent generation clinical CT scanner. As awareness of their existence spreads and as technological capabilities continue to improve, it is likely that the detection and recognition of these lesions in vivo will become more frequent. 

In our study, we optimized a procedure for histological evaluation of tissues. To our knowledge, ours is the first to assess human osteochondral tissues in this manner. Specifically, relatively thick (1–2 mm) sections were used, whereas traditional histological sections for brightfield or fluorescence imaging are typically <10 µm thick. We posit that our procedure facilitated the identification of small vessels since each side of the section could be imaged up to 250 µm in depth. Not only was more tedious serial sectioning avoided, but an adequate amount of tissue was left undisturbed within the section and free from sectioning artifact. Moreover, our pipeline avoids embedding in plastic or paraffin and greatly shortens bone decalcification time (which was permissible at 4 °C), all of which optimize the preservation of enzymatic activity and antigen integrity [32,33]. The procedure herein described is also compatible with subsequent immunostaining as well as tissue clearing protocols, such as iDISCO [34]. Finally, the thicker histological tissue sections and z-stack images facilitated comparison with clinical imaging modalities, such as CT and MRI. 

Our histological findings agree with existing reports, wherein articular cartilage undergoes changes in vascular channel density, in parallel with disease state. Walsh et al. report a bi-directional change in channel density, involving an increase in tidemark-crossing channels in OA, as well as a decrease in the total density of channels, relative to controls [35]. Without subdividing our tissue areas with respect to HDMP status, our data is consistent with the total sum of channels reported by Walsh et al. in their study of OA, and with their mean and ranges terminating in cartilage below and above the tidemark. When we consider HDMP-containing and HDMP-free areas, we observe significant changes in the distribution of channel density and in the density of channels that terminate in the uncalcified cartilage with the same two directions of change Walsh reported in OA. Taken together, these results suggest a continuum of disease severity for which the changes in total and tidemark-crossing vascular channel distributions may be used as a marker.

The high density of deeply penetrating vascular channels in OA and especially in the HDMP-containing areas may be etiologically significant to OA progression, or may be a consequence of disease progression. Channels are connected to marrow spaces and are believed to be constructed by osteoclasts followed by or in concert with vascular invasion [35,36]. Indeed, we observed high TRAP activity in the vascular channels. The overall lower occurrence of channels in OA and especially in HDMP-containing areas combined with the lack of widespread increase in TRAP activity is inconsistent with only a large-scale non-specific upregulation of bone-resorptive activity as might occur in response to bone health-related changes in diffusible factors [37]. Instead, a more local change likely encourages extension of shallow channels across the calcified cartilage. Given that cartilage-resorptive activity has been localized to the endothelial cells of growing H-type vessels rather than to their co-migrating osteoclasts [38], some angiogenesis-promoting factor may be present at locally higher concentrations in HDMP-containing regions, driving the lengthening of channels into the uncalcified cartilage. The known aging- and disease-associated decrease in the molecular weight of matrix glycosaminoglycans may provide such a candidate [37]. Low-molecular-weight degradation products of matrix hyaluronic acid stimulate angiogenesis [39], and the specific role of these fragments in bone and cartilage homeostasis is under investigation [40]. Interestingly, previous studies on vascular calcification have shown that undegraded high-molecular-weight hyaluronic acid may suppress the dystrophic calcifications that are promoted by BMP-2 signaling [41,42,43,44]. BMP-2 is associated with production and maintenance of healthy cartilage [45,46,47,48,49,50], and is upregulated in OA and cartilage damage [51,52,53,54], presumably as a reparative response. Thus, dystrophic calcification in the form of HDMPs may depend on the combination of chronically diminishing protective effects of a normal hyaluronic acid-rich matrix and excessive BMP-2 activity, facilitating a pathological acute response to osseous micro-cracks or other initiating events.

Our study has several limitations. First, we had a limited sample size, and tissues were obtained from subjects with advanced osteoarthritis. Future research should focus on a larger number of patients with earlier stages of osteoarthritis. Second, our subjects were all male. This was due to the bias of the institution at which the study was performed and is not representative of typical osteoarthritis populations, particularly since the disease is actually more common in women [55]. Third, our study was cross-sectional in nature, and while we demonstrated an association between HDMPs and COs, our study design is insufficient to elucidate the complete timeline of these lesions. It remains likely that HDMPs may progress along a variety of different paths, including extrusion into the joint cavity or resorption without ossification, and that COs may develop in the absence of an HDMP. Longitudinal in vivo imaging studies, particularly with the impressive continuing advancements with in vivo imaging technology, will be required to provide these answers. Fourth, HDMPs were strictly defined in our study and identified using clinical CT imaging. Mineralized protrusions that were of lower attenuation (e.g., iso-attenuating to surrounding cartilage) or smaller than the resolving capability of our CT scanner may not have been detected. Again, future studies employing new technologies are likely to improve on this, such as the use of photon-counting CT scanners, which provide a higher contrast-to-noise ratio, improved spatial resolution, and optimized spectral imaging relative to current CT technology [56]. Finally, the biochemistry of HDMPs was not assessed in this study and remains to be elucidated. However, previous studies have suggested a strong morphologic similarity with dystrophic soft tissue calcification, in particular those regions where mineralization occurs in locations without pre-existing cells and or abundant extra-cellular matrix components [20,57]. 

## 5. Conclusions

In conclusion, HDMPs and COs are associated lesions in patients with advanced knee osteoarthritis. They are common and may be identified using clinical imaging modalities. HDMPs and COs share similar features, including increased vascularization and metabolic bone activity at the OCJ. Future studies are needed to determine the relationship of these lesions with osteoarthritis progression and symptomatology. Increasing awareness of the existence of these lesions with knowledge of their various appearances are appropriate first steps. 

## Figures and Tables

**Table 1:** Patient demographics and clinical data.

Patient	Age	Gender	Side	BMI	Hypertensive	Smoker
1	68	M	L	32.2	+	+
2	84	M	L	28.0	+	-
3	73	M	R	23.8	-	-
4	66	M	L	32.4	+	-
5	66	M	L	26.3	-	-
6	67	M	R	29.6	+	+
7	67	M	L	31.3	-	-
8	70	M	L	26.4	+	-
9	72	M	R	24.4	+	-
10	65	M	L	37.6	+	-
11	77	M	L	35.4	+	-
12	72	M	R	28.9	-	+
13	71	M	L	25.7	-	-
14	58	M	R	27.6	+	-
15	63	M	L	24.4	+	-
16	66	M	R	26.9	+	-
17	50	M	L	27.7	-	-
Mean (±SD)	67.9 ± 7.4			28.7 ± 3.9		

SD: Standard deviation; BMI: Body mass index.

**Table 2:** HDMPs and central osteophytes distribution among patients.

Patient Number	HDMP	Central Osteophyte
1	-	-
2	+	+
3	+	+
4	-	-
5	-	-
6	-	-
7	-	-
8	-	-
9	+	-
10	+	+
11	+	+
12	-	-
13	+	+
14	+	+
15	-	+
16	-	-
17	-	-

Fisher’s exact test *p*-value: 0.003. HDMP: High-density mineralized protrusions.

## Abbreviations

HDMP	High-Density Mineralized Protrusion
CO	Central Osteophyte
OCJ	Osteochondral Junction
DAPI	4′, 6-diamidino-2-phenylindole
ELF	Enzyme labeled fluorescence
UEA	Ulex europaeus agglutinin 1
TRAP	Tartrate-resistant acid phosphatase activity type 5
iDISCO	Immunolabeling-enabled imaging of solvent-cleared organs
CT	Computed Tomography
MRI	Magnetic Resonance Imaging
BMP-2	Bone morphogenetic protein 2
